# Laboratory Studies on the Rheotaxis of Fish under Different Attraction Flow Conditions

**DOI:** 10.3390/ijerph19095744

**Published:** 2022-05-09

**Authors:** Nanbo Tang, Xiaogang Wang, Yun Li, Long Zhu, Zhushuan Tang, Hongze Li, Feifei He, Yongzeng Huang, Zhengxian Zhang

**Affiliations:** 1 State Key Laboratory of Hydraulics and Mountain River Engineering, Sichuan University, Chengdu 610065, China; tangnanbo@stu.scu.edu.cn; 2 State Key Laboratory of Hydrology-Water Resources and Hydraulic Engineering, Nanjing Hydraulic Research Institute, Nanjing 210029, China; zhulong@nhri.cn (L.Z.); lihz0227@163.com (H.L.); ffhe@foxmail.com (F.H.); hyz_tzs@163.com (Y.H.); 3 Anhui Provincial Group Limited for Yangtze-to-Huaihe Water Diversion, Hefei 230601, China; terrtang@foxmail.com; 4 State Key Laboratory of Water Resources and Hydropower Engineering Science, Wuhan University, Wuhan 430072, China; zhangzx@whu.edu.cn

**Keywords:** rheotaxis, attraction flow, fish passage entrance, river restoration

## Abstract

The damming of the river changes the structure of the original river ecosystem, and although fish passage plays an important role in maintaining the connectivity of the river ecosystem, the fish have difficulty finding the fish passage entrance during the upstream process. This paper studied the rheotaxis of fish under three different water flow conditions experimentally through recirculating water tanks. To better understand the response of Crucian carp (*Carassius auratus*) to water flow stimulation, the representative swimming trajectory, sensing success rate, attraction success rate, reaction time, and attraction time of the fish were analyzed by using a video monitoring system. The experimental results showed that fish responded differently to single-peak and lateral bimodal outflow conditions: (1) the single-peak outflow condition had a much better attraction effect than the lateral bimodal outflow condition, both in terms of sensing success rate and attraction success rate; (2) the fish swam mainly in the middle area of the lateral bimodal outflow condition, while the fish swam more evenly in the single-peak outflow condition. Therefore, setting the attraction current at the right time and near the entrance of the fish passage may help to improve the effect of fish attraction.

## 1. Introduction

China is the country with the largest number of dams in the world; according to statistics, 98,000 dams have been built, and there are more than 270,000 water conservancy facilities, such as gates and weirs, with a discharge of 1 m^3^/s and above [1]. While promoting the benefits and eliminating the harm, these dams and reservoirs also directly block the connectivity of river water flow, destroy the topography and geomorphology of the original river, make the aquatic environment of the river discontinuous, artificially cut off the channels for fish and other aquatic organisms to migrate, resulting in the loss of fish population diversity and even the endangerment of long-distance migratory fish. It is urgent to restore the longitudinal connectivity of rivers and fish migration channels. Fish passage, which contributes to the restoration of river connectivity and helps fish to complete their upstream migration, is gaining a lot of attention.

The earliest fish passages were excavations, reefs in the river, dredging rapids, and other natural obstacles to communicating the migratory route to the fish [2,3,4,5]. In 1662, France’s Bayonne province issued regulations [6] requiring the construction of weirs on the passage of fish upstream and downstream. However, the structure of this fish passage was simple—only some branches were fixed at the bottom of the weir to reduce the flow of water, allowing fish to pass the weir. The world’s first real fish passage [7,8] was built at the Hooley Dam on a tributary of the River Tees in Perthshire, Scotland in 1883. By the end of the 1990s, more than 400 fish passages had been built in North America and more than 1400 in Japan [9,10]. The study of Chinese fish passages began in 1958, relatively late compared to foreign countries [11]. For a long time afterward, the development of hydropower projects in China became the focus, neglecting the protection of the ecological environment. In the growing pursuit of sustainable and harmonious development, China has placed a high priority on the protection of the ecological environment since November 2012. For the protection of fish and other aquatic plants and animals, additional fish passages and stocking measures have been proposed one after another [12,13,14]. The “European Water Framework Directive” also requires the restoration of river continuity [15] through the construction of efficient fish passages by 2027.

At present, most fish passage studies have been devoted to optimizing the structural design of fish passages to improve the passing capacity [16,17,18]. Many researchers such as Amaral [19], Baki [20], Enders [21], and Shicheng Li [22] have also studied the swimming performance and appropriate flow velocity of fish in the interior of the fish passages. Starrs [23] used burst swimming speed to predict the ability of fish to pass fish passages and proposed that water temperature and flow velocity are two important factors for fish to pass fish passages. Hein [24] studied a variety of migratory animals and proposed a model to calculate the maximum migration distance, which is closely related to the size of the animal. Foreign scholars have obtained various evaluation models [25,26] of swimming ability through a large number of studies, such as the relationship between swimming speed and body length; the relationship between swimming speed, body length, and fatigue time; the relationship between temperature and swimming speed; the relationship between temperature, body length, and swimming speed [27,28]; and the relationship between swimming speed and oxygen consumption rate [29]. Yang Yu [30] introduced the research of fish hydraulics from three aspects, including the demand of fish for water environments, the physiological tolerance of fish, and the ecological tolerance of fish, and suggested further exploring the action law and mechanism of water flow in the ecological problems of fish and developing the hydraulics of overfishing facilities and fish habitat restoration.

Green [31] found that fish tend to be attracted to the rapids of hydroelectric power plants, which makes it difficult for fish to identify fishway entrances. Because the water volume released from the fishway is relatively low, the velocity of flow is usually extremely slow. Therefore, the attractiveness of the fish passage entrance is also critical to ensure that the fish passage is fully functional. According to the “Fishway guidelines for Washington State” [32], the number of fishways that can effectively pass fish is generally below 50% in countries around the world, and the ability of fish to quickly find and accurately enter the fishway entrance is one of the key factors in the successful operation of fishways. However, a part of the reason for the low efficiency of fish passage is that it is difficult for target fish to find the entrance of the fish passage during the upstream process [33]. Xie Chunhang [34] used numerical simulations to study two types of fish passage entrance arrangements and found that the flow pattern of the fish-catching water in the fish-catching channel formed by the guide wall and the river bank was more attractive to the target fish. Fishway entrances belong to the intersection of fish behavior and hydraulics research; however, most of the current literature mentions fishway entrance design from the engineering operation point of view and hydraulics. In this paper, we studied the rheotaxis of experimental fish under different water flow conditions through an indoor tank to create fish-friendly water flow conditions for fish passage projects and improve the fish collection and lure effect of fish passage entrances.

## 2. Materials and Methods

### 2.1. Experimental Setup

**Figure 1:**
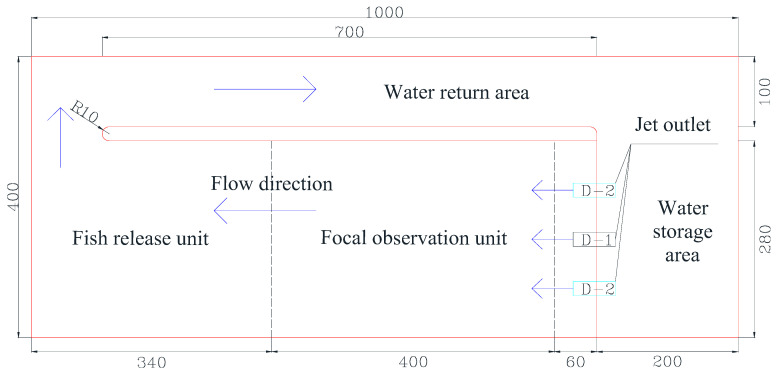
Test model generalization diagram (unit: cm) (D-1—it represents the flow conditions of single-peak outflow; D-2—it represents the flow conditions of bimodal outflow). The red line is the boundary line of the model (actually exists), and the black dotted line is the schematic line of the division of the internal area of the model (not really exists).

**Figure 2:**
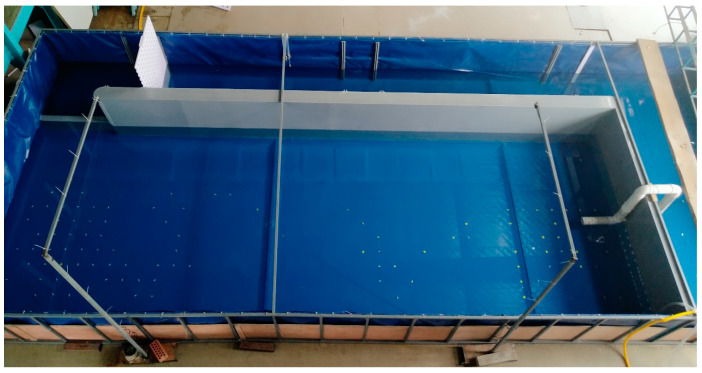
Experimental model photo.

The fish experiments were conducted in an indoor self-built tank at the Tiexinqiao Water Experiment Center of the Nanjing Institute of Water Resources Science from May to September 2021. The self-built tank was a circular self-circulating tank with a total length of 10.0 m, a total width of 4.0 m, and a total height of 1.0 m. The tank was divided into three areas, namely the water storage area, the test area, and the water return area, by using a gray plastic baffle (Figure 1). The water pump was used to extract the water stored in the storage area into the test area, and the water flowing out of the test area entered the storage area through the return channel to achieve self-circulation of the water supply. Before the experiment, the effect of the 1.0 m wide return channel was tested, and it was found that it could quickly and effectively replenish the reduced water in the storage area and ensure that the pump provided the experimental water stably. To facilitate the analysis of the experimental results, the effective test area of 7.0 m in length and 2.8 m in width was divided into two units: the fish-release unit (downstream side) and the focal observation unit (upstream side). The fish release unit was 2.4 m long and 2.8 m wide, and was used to simulate the habitat area of downstream fish; the effective test area 4.0 m long and 2.8 m wide was the focal observation unit. The path chosen by the experimental fish to move from the fish release unit to the upstream water flow outlet area was completely recorded by a camera arranged above the tank. The camera used for the experimental video recording was a custom-made HD camera (sensor was Sony IMX317(1/2.5”), the resolution and frame rate were 3840 × 2160@30 fps, FOV was 100 degrees, relative illumination (sensor) was 70%, and IR filter was 650 ± 10 nm). To minimize the distortion of the image during the video recording, the camera was fixed on a high enough bracket to ensure that the fish movement could be observed in all the experimental areas of the tank (Figure 2).

### 2.2. Target Fish and the Acclimation Conditions

**Figure 3:**
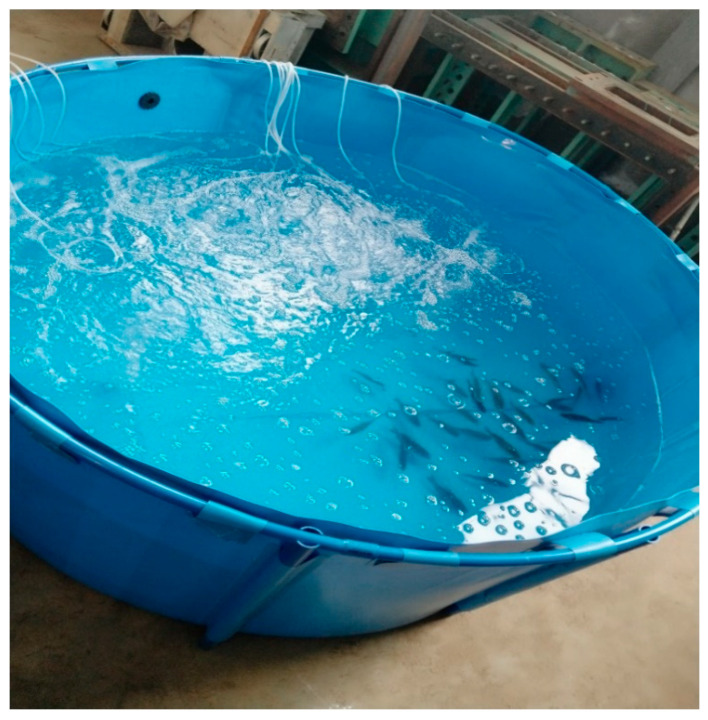
Experimental fish in the temporary storage tank.

Crucian carp (*Carassius auratus*) was selected as the target fish in this study. *Carassius auratus* is a typical freshwater fish native to China, widely distributed in all major water systems outside the Qinghai-Tibetan Plateau region of China, and it has been introduced to freshwater bodies around the world. The wild population mainly inhabits lakes, rivers, canals, and marshes, especially in shallow lakes with lush water. It is an adaptable fish that can survive in deep or shallow water, running or still water, or high or low temperature water, and even in alkaline water and saline lake Dali, it can still grow and reproduce. The *Carassius auratus* used in the experiment were provided by a local fish farm in Jiangsu, China. To avoid harming the fish, the fish were not caught by electric fishing, but by less harmful fishing nets, and then they were kept in a portable container with an oxygenator. The fish were then transported to the Tiexinqiao Water Experiment Center of the Nanjing Institute of Water Resources Science in less than two hours to adapt to the laboratory environment. To minimize the adverse effects [35] of stress on the subsequent experiments, the weight and sex of the fish were not collected, but only the length of the fish was measured. The local fishery provided 50 individuals of cultured Carassius auratus with an average size of about 18.8 ± 0.4 cm. In the pre-test, it was observed that the target fish were able to swim at burst speeds in the test area, and therefore, the size of this test area was considered to meet the needs. All experimental fish were temporarily housed for 5 days in a circular pool of 2.0 m diameter in the laboratory at a depth of 0.6 m (Figure 3). Temporary water was tap water that had been aerated for 3 days, with a water temperature of 23 ± 1 °C and a dissolved oxygen concentration greater than 8.0 mg/L. These fish were regularly fed with appropriate fish food, and feeding was stopped one day before the experiment. For the formal experiment, the experimental water was the same as the temporary water, with a water temperature of 24 ± 0.5 °C and dissolved oxygen concentration maintained above 8.0 mg/L.

### 2.3. Experimental Setup

To study the convergent behavior of fish under different water flow conditions, 15 sets of experiments containing three different conditions were conducted in the laboratory, namely, the lateral symmetric bimodal outflow mode, lateral asymmetric bimodal outflow mode, and single-peak outflow mode (Table 1). In the lateral symmetric bimodal outflow mode, the flow rate was set to 80 m^3^/h, i.e., the flow rate of each outlet was 40 m^3^/h. The water depth was maintained at about 40 cm by adjusting the sink tailgate. The two outlets were arranged at 1/4 of the width from the lateral sidewalls of the test area (70 cm from the side walls). In the lateral asymmetric bimodal outflow method, the positions of the two outlets were kept the same as in the lateral symmetric bimodal outflow method for comparison purposes. The discharge was also set to 80 m^3^/h, and the discharges of the two outlets were set to 65 m^3^/h (left) and 15 m^3^/h (right) for one large and one small outlet, respectively. The cross-sectional shape of the spout with a circular shape and its ejected discharges of 15 m³/h, 40 m³/h, and 65 m³/h were provided by calibrated pumps. The diameters of their spouts were 14 cm, 7.5 cm, and 6.0 cm, respectively. In the single-peak outflow mode, the discharge was set to 65 m^3^/h and the flow outlet was set at the 1/2 width of the test area. To reasonably determine the vertical position of the outlet, the flow conditions of surface outflow (at 30 cm above the bottom) and bottom outflow (at 10 cm above the bottom) were compared. The test results showed that: under the bottom outlet condition, the mainstream swung obviously due to the influence of the bottom sidewall, and the flow pattern was chaotic, which had a great influence on the swimming behavior of the experimental fish; under the surface outlet condition, the mainstream developed downstream in a straight line, and there was no obvious lateral swing, and the flow pattern met the required requirements of the test. Therefore, the vertical position of the outlet of the experiment under three different water flow conditions was arranged at 30 cm above the bottom, and the experimental water depth was maintained at about 40 cm.

### 2.4. Analysis of Fish Behavioral Response

The experiment was conducted from May to September, and the experiment was chosen to be conducted indoors to avoid excessive temperature differences due to sunlight. For each experiment, a dozen of experimental fish in the temporary pond were randomly caught with soft fishing nets, the fish were gently placed into the release unit by manual netting method, and the experimental fish were allowed to acclimatize in the release unit until they stopped swimming freely before starting the test (about 30 min) [35]. The swimming behavior of the experimental fish was recorded by the monitoring system. If the experimental fish successfully swam from the release unit into the focal observation unit, the experimental fish were considered to have successfully sensed the stimulus of water flow; if the experimental fish successfully swam from the release unit to the vicinity of the outlet, the experimental fish were considered to be successfully attracted. The maximum observation time for each group of tests was 40 min, and if the experimental fish failed to reach the vicinity of the water outlet after 40 min, the experimental fish were considered not successfully attracted. Nevertheless, such a short adaptation time and duration of the experiment may have caused some variation in the results [36,37]. To avoid the effects of light and time, the experiments were completed during well-lit periods, and the time of the experiments was kept the same as much as possible. At the end of the experiment, the fish were transferred to another rectangular transient pond through the experimental basin to observe whether there were any casualties, and after 24 h, the fish without obvious trauma were transferred to the circular transient pond. Since the individual fish were not marked, some of the experimental fish randomly caught for each group of experiments may have been tested more than once, and it was also difficult to record the number of times each individual fish was used. Therefore, multiple experimental tests of some fish may have affected the experimental results, but this effect proved to be likely insignificant [38].

To better characterize the rheotaxis of experimental fish under different water flow conditions, here, we first defined four parameters: sensing success rate, attraction success rate, reaction time, and attraction time.

If the experimental fish successfully swam from the fish release unit into the focal observation unit, the experimental fish were considered to have successfully sensed the stimulation of the water flow, and the sensing success rate was calculated for each group of tests. The sensing success rate was calculated by the formula:

(1) $$S_{S}=\frac{N_{S}}{N_{N}}\times 100\%$$

where $N_{S}$ is the number of experimental fish that successfully swam from the fish release unit into the focal observation unit in each group of tests; $N_{N}$ is the total number of fish released in each group of tests.

If the experimental fish successfully swam from the release unit to the vicinity of the outlet, the experimental fish were considered to be successfully attracted, and the attraction success rate was calculated for each group of tests. The attraction success rate was calculated by the formula:

(2) $$S_{A}=\frac{N_{A}}{N_{N}}\times 100\%$$

where $N_{A}$ is the number of experimental fish that successfully swam from the fish release unit into the vicinity of the water outlet in each group of tests; $N_{N}$ is the total number of fish released in each group of tests.

Timing from the jet discharge of the water outlet, the time until the experimental fish entered the focal observation unit from the release unit was defined as the reaction time $T_{R}$.

The time spent by the experimental fish passing through the focal observation unit was defined as the attraction time $T_{U}$.

### 2.5. Test and Measurement Means

Considering that the water depth in this test was only 40 cm, the water depth in the test area of the flume varied in a small range, and the water pressure did not vary much along with the depth, which also did not easily cause significant changes in fish behavior in the vertical direction. At the same time, based on the qualitative test observation in the early stage, it was found that the fish entering the tank quickly found their favorite water depth and kept this depth upstream, which indicated that the movement trajectory of fish in this experimental environment mainly changed in the plane. Therefore, this experiment mainly collected the motion trajectories of experimental fish in the two-dimensional XY plane. The experiment used Logger Pro 32 software to track the fish movement, which uses the number of video frames as the time axis and the pixel point assignment to track the target, and can capture the frames in the video frame-by-frame to track the process of the target movement. By importing the captured video into this software and playing back the video, the fish’s swimming can be tracked frame-by-frame. Assuming that the shape and size of the fish have almost no effect on the path extraction, the trajectory is characterized by the movement of the fish’s head to generate the fish’s motion trajectory. At the end of the playback of the whole video tracking, the software generated a data table containing the coordinate point positions of the experimental fish on the horizontal plane at different moments and the swimming speed of the fish in the horizontal direction.

To understand the flow velocity in the focal observation unit more quantitatively, a three-dimensional ADV velocity measurement system and a wireless propeller velocity meter were used to measure the flow field in the test area. We laid 18 flow measurement sections (section spacing 0.20 m) in the focal observation unit. We evenly distributed 15 measurement points (measurement point spacing 0.20 m) in each flow measurement section. A total of 270 measuring points were set up, and the water depth at the measuring point was 0.30 m. The ADV velocity measurement system uses an acoustic Doppler velocimeter Vectrino (Nortek, Akershus, Norway). Flow velocities were collected at 25 Hz for 2 min at each measurement location. The selection of velocity range must be greater than the velocity value of the measuring point (the measurement effect is the best if it is slightly greater). The wireless propeller velocity meter is a miniature contact intelligent velocity meter (LGY-Ⅲ) developed by Rui Di High-Tech Company of Nanjing Institute of Water Resources Science, Nanjing, China, which was pre-calibrated by ADV before the experiment. It was mainly used for the hourly average discharge measurement in the laboratory. The water temperature of the test water was measured by a high-precision mercury thermometer commonly used in laboratories, with a measurement accuracy of ±0.2°. The dissolved oxygen concentration measurement in water was adopted by the HACH HQ30d (HACH, Loveland, CO, USA) dissolved oxygen meter imported from the USA, and the accuracy of dissolved oxygen was ±0.1 mg/L for 0.1–8.0 mg/L and ±0.2 mg/L for greater than 8.0 mg/L. One-way analysis of variance (ANOVA) was performed using the SPSS Statistics software (IBM V21.0; IBM, New York, NY, USA) to assess the significant effects. The statistical significance of the results was accepted as *p* < 0.05 in all tests performed.

## 3. Experimental Results

### 3.1. Swimming Mode

**Figure 4:**
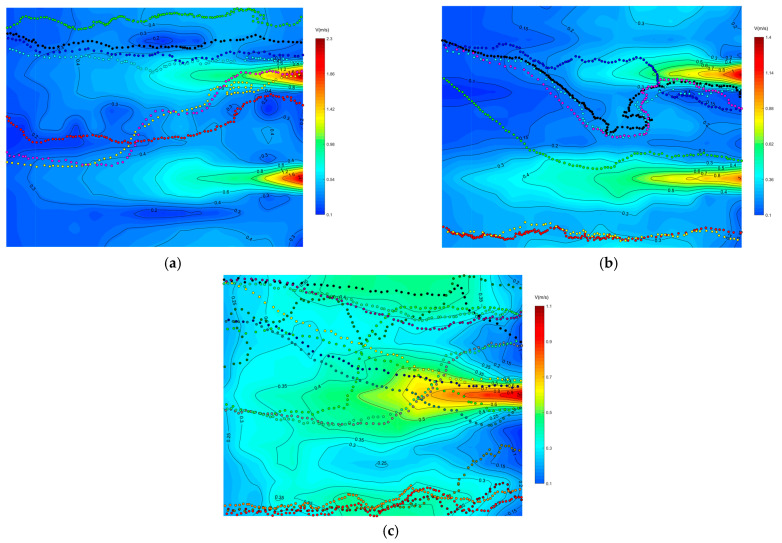
Swimming trajectory of some experimental fish in different water flow conditions. (**a**) Lateral symmetric bimodal outflow; (**b**) Lateral asymmetric bimodal outflow; (**c**) Single peak outflow.

**Figure 5:**
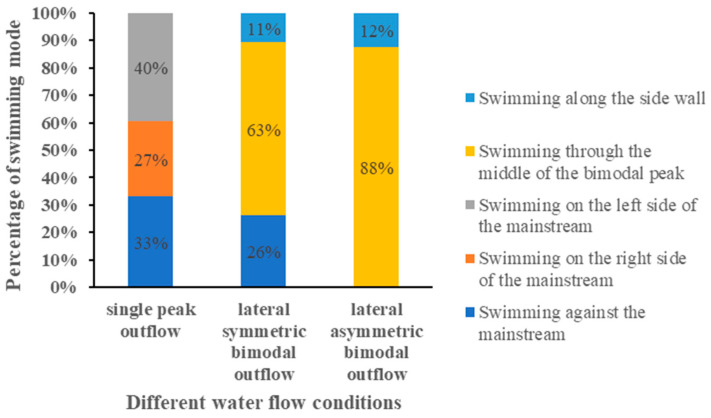
The percentage of swimming mode under different water flow conditions.

Figure 4 shows the swimming trajectory of some experimental fish under different water flow conditions. Figure 5 shows the percentage of swimming mode under different water flow conditions. From the figure, it can be seen that the swimming trajectory of the experimental fish under lateral symmetric bimodal outflow condition could be divided into three swimming modes: swimming through the middle of the bimodal peak, swimming against the mainstream, and swimming along the sidewall. The total number of experimental fish was 48, and the number of successfully attracted fish was 19; the swimming mode appeared in a larger proportion of the situation, and the swimming mode was more obvious, mainly through the middle of the bimodal peak swimming, accounting for 63%; swimming against the mainstream accounted for 26%; and along the sidewall swimming accounted for 11%. The total number of fish tested in the lateral asymmetric bimodal outflow condition was 92, and the number of successfully attracted fish was 32. Swimming through the middle of the bimodal peak accounted for 88%; swimming along the sidewall accounted for 12%; the experimental fish swam in a relatively single direction, and an obvious single swimming mode appeared. The swimming mode of the experimental fish in the lateral asymmetric bimodal outflow condition was different from the lateral symmetric bimodal outflow condition in that the swimming against the mainstream mode did not appear. The total number of fish in the single-peak outflow condition was 80, and the number of successfully attracted fish was 66. The percentage of swimming against the mainstream was 33%; the percentage of swimming on the right side of the mainstream was 27%; and the percentage of swimming on the left side of the mainstream was 40%. The swimming mode of the experimental fish was relatively average, and there was no obvious single swimming mode. The swimming mode could be divided into three swimming modes: swimming against the mainstream, swimming on the right side of the mainstream, and swimming on the left side of the mainstream. Here, the classification of the swimming mode under the three water flow conditions is partially supplemented. The experimental fish swimming through the high-velocity zone of the mainstream into the vicinity of the outlet was classified as swimming against the mainstream, and the experimental fish swimming from the low-velocity zone on both sides of the mainstream into the vicinity of the outlet was classified as swimming on the right side of the mainstream and swimming on the left side of the mainstream. The experimental fish were classified as along the sidewall swimming when they swam away from the mainstream and always on the sidewall.

### 3.2. Visualization of the Upstream Path

**Figure 6:**
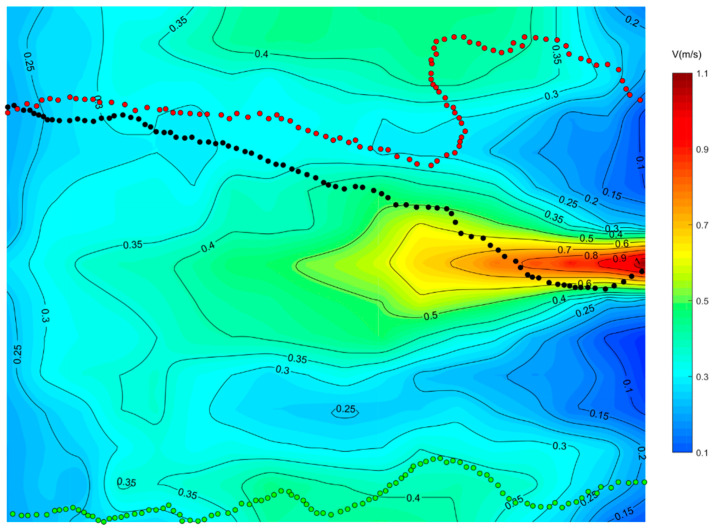
Superposition of representative travel trajectory and flow field in a single-peak outflow condition focused observation cell.

**Figure 7:**
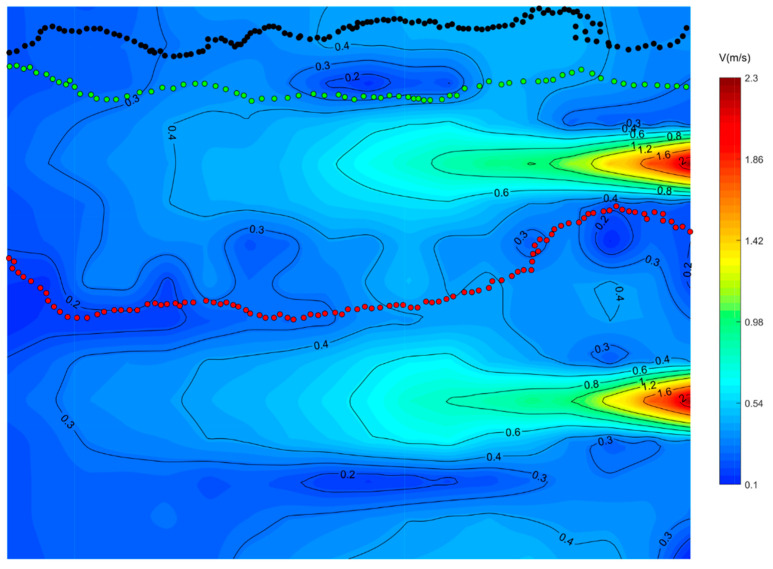
Superposition of representative swimming trajectory and flow field in the focused observation cell for the lateral symmetric bimodal outflow condition.

**Figure 8:**
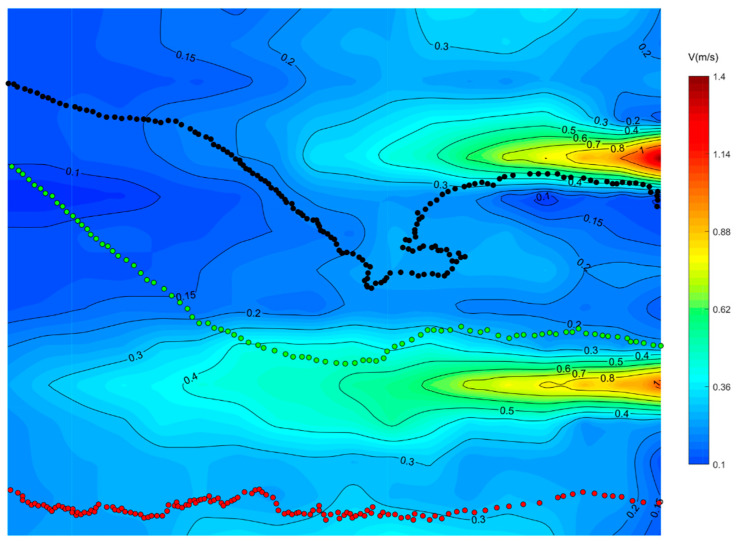
Representative swimming trajectory and flow field superposition in the focused observation cell under the lateral asymmetric bimodal outflow condition.

The representative swimming trajectories of experimental fish in single-peak outflow, lateral symmetric bimodal outflow, and lateral asymmetric bimodal outflow conditions with superimposed flow fields are shown in Figure 6, Figure 7 and Figure 8, respectively. The selection of representative trajectories was mainly based on the motion observed in this experiment, which was divided into three principles. The first principle was to select the shortest length of the trajectory under the water flow condition as the representative trajectory. The second was to select the trajectory with a relatively special motion under the water flow condition, such as the trajectory with a 90° or 180° turn. The third was the trajectory with the highest frequency (including similar trajectories and the same trajectory brought by group motion). In the figures, the direction of the water flow was from the right (upstream) to left (downstream), while the fish swam in the opposite direction.

As can be seen from the Figure 6, the mainstream of the single-peak outflow condition basically flowed downstream along a straight line, and the mainstream gradually spread to both sides along the way, forming a vortex on both sides of the mainstream, but the intensity of the vortex on both sides was not large, the size of the flow velocity was basically in the range of 0.30~0.40 m/s, and the experimental fish did not lose their way in the vortex. Path 1 (black dots) appeared as high as 44%, and the path of the experimental fish in the front two-thirds of the focal observation unit was mainly in the flow velocity range of 0.20~0.50 m/s. The experimental fish moved along an inclined straight line and gradually approached the core mainstream area above the flow velocity of 0.50 m/s upstream. In the one-third area behind the focal observation unit, the experimental fish swam through the core high-velocity mainstream and entered the jet outlet area. The different path 2 (red dots) accounted for 32%, and the path of the experimental fish in the front two-thirds of the focal observation unit was parabolic with a small amplitude. The experimental fish did not approach the central mainstream in the latter third of the focal observation unit but swam to the jet outlet area by turning 90° to the sidewall area where the flow velocity was low (0.30~0.40 m/s) and the flow pattern was smooth.

It is important to emphasize the representative path 3 (green dots), which occurred in only 11% of the experiments but was present in the rest of the two bimodal outflow conditions. The experimental fish swam mainly along the left (or right) sidewall in the low-flow velocity zone of 0.20~0.40 m/s throughout the focal observation unit and swam the whole focal observation unit with the path away from the central mainstream. The flow velocity under this path was relatively small and the flow pattern was smooth, which was one of the typical swimming trajectories of fish.

As shown in Figure 7, under the condition of lateral symmetric bimodal outflow, the smooth flow pattern of the two mainstreams, with a small swing from left to right appeared. A 40~50 cm wide low-velocity reflux zone (0.2~0.4 m/s) appeared in the middle region of the two streams. The percentage of path 2 (green dots) was 32%. The path of the experimental fish in the focal observation unit was basically along the outer edge of the right stream, and the flow velocity of the path was mainly in the range of 0.20~0.40 m/s. The experimental fish passed through some small eddies of low velocity and swam gradually to the jet outlet area. This path successfully avoided the area of maximum flow velocity, which was one of the preferred swimming paths for fish. Path 3 (red dots) appeared in a high percentage of 48%, fish were attracted by the flow and gradually approached the middle area of the bimodal peak along an inclined straight path, and the flow velocity of this path was basically around 0.20 m/s. After that, they gradually moved away from the left stream and entered the low flow velocity area between the two streams, and the return flow velocity of this path was mainly 0.20~0.40 m/s.

As shown in Figure 8, in the lateral asymmetric bimodal outflow condition, due to the large difference between the flow of the two streams resulting in the secondary mainstream on the right (15 m^3^/h) downstream to a much smaller distance than the left mainstream (65 m^3^/h), which in turn formed a whirlpool at the end of the right stream, the width of the whirlpool was about 80~100 cm, and the size of the flow velocity value was 0.05~0.20 m/s, which did not make the test fish lost in the vortex. Path 1 (black dots) appeared in 38%, the experimental fish were attracted by the flow, the flow velocity in the front third of the path of the focal observation unit was below 0.20 m/s, and gradually approached the secondary mainstream on the right (15 m^3^/h); the flow velocity for one-third of the path after passing through the right mainstream into the middle low-flow area of the two streams was between 0.20 m/s and 0.30 m/s; and after encountering the vortex in the middle low-velocity area of the two streams, the experimental fish first turned 180° and then turned 90° to find the swimming path toward the secondary mainstream on the right (15 m^3^/h), and gradually swam along the left outer edge of the right stream to the jet outlet area. The different path 2 (green dots) accounted for 34%, the flow velocity of the experimental fish in the front one-half of the path of the focal observation unit was below 0.20 m/s, fish gradually swam to the low-flow velocity area between the two streams, and this section of the path was a straight line; after that, the fish were attracted by the high-flow velocity of the left mainstream (65 m^3^/h) in the middle low-flow velocity area, and it swam to the right outer edge of the left mainstream (65 m^3^/h) in the 0.40 m/s flow velocity area; and the swimming path in the second half of the focal observation unit was along the right outer edge of the left mainstream (65 m^3^/h) where fish swam to the jet outlet area in a straight line, and the flow velocity in this section of the swimming path was between 0.20 m/s and 0.50 m/s.

### 3.3. Sensing Success Rate and Attraction Success Rate

**Figure 9:**
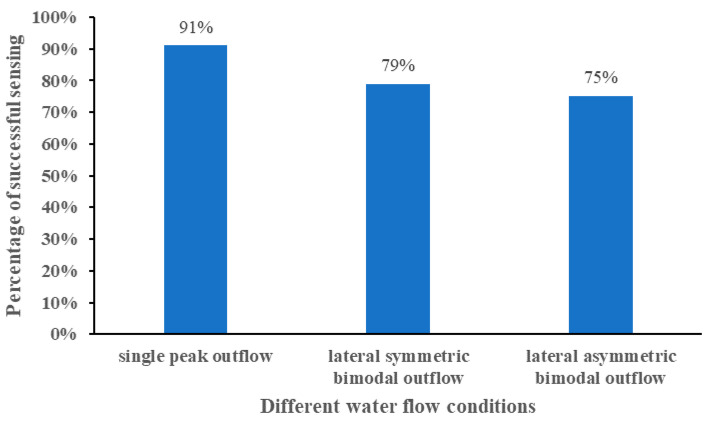
Average sensing success rate under different water flow conditions.

The average sensing success rate under different water flow conditions is shown in Figure 9. The ANOVA results are tabulated in Table 2. Under the three water flow conditions, the experimental fish had the best effect on the water flow stimulation under the single-peak outflow condition, with an average sensing success rate of 91%. Under the bimodal outflow condition, the test fish sensed the water stimulation effect of the lateral symmetric bimodal outflow condition and the lateral asymmetric dual-peak outflow condition equally, and the average sensing success rate was 79% and 75%, respectively. Although the discharge under the single-peak outflow condition was smaller than that under the bimodal outflow condition, the fish sensing success rate under this condition was better than that under the bimodal outflow condition, and the average sensing success rate of the three water flow conditions was single-peak outflow > lateral symmetrical bimodal outflow condition > lateral asymmetrical bimodal outflow condition.

**Figure 10:**
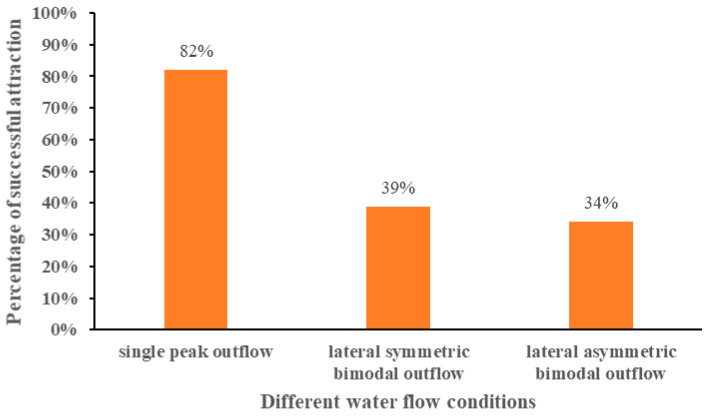
Average attraction success rate under different water flow conditions.

The average attraction success rates of different water flow conditions are shown in Figure 10, and the ANOVA results are tabulated in Table 2. The experimental fish under the single-peak outflow condition showed the best attraction effect, with an average attraction success rate of 82%, even when the attraction discharge of 65 m^3^/h under the single-peak outflow condition was smaller than the attraction discharge of 80 m^3^/h under the bimodal outflow condition; under the bimodal outflow condition, the average attraction success rate of the lateral symmetric bimodal flow and the lateral asymmetric bimodal flow were lower, at 39% and 34%, respectively, which was much smaller than that of the single-peak flow condition; the average attraction success rate of the three water flow conditions was single-peak flow > lateral symmetric bimodal flow condition > lateral asymmetric bimodal flow condition.

### 3.4. Reaction Time and Attraction Time

**Figure 11:**
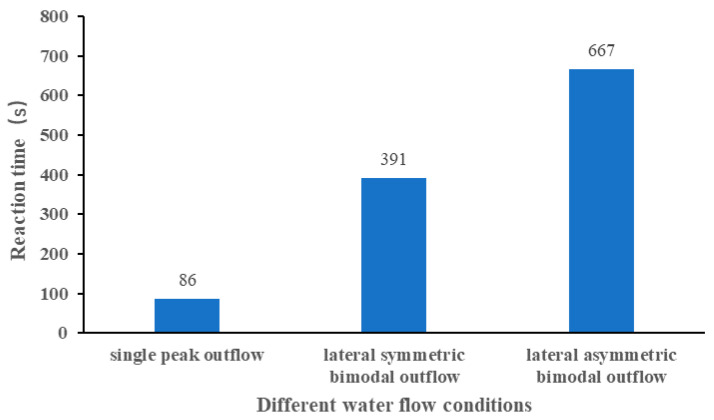
Average reaction time under different water flow conditions.

The average reaction times of the experimental fish for the single-peak outflow, lateral symmetric bimodal outflow, and lateral asymmetric bimodal outflow conditions were 86 s, 391 s, and 667 s, respectively, as shown in Figure 11. The ANOVA results are tabulated in Table 3. The shortest average reaction time was 86 s, which appeared in the single-peak outflow condition, and the reaction time was much smaller than in the bimodal outflow condition; the longest average reaction time was 667 s, which appeared in the lateral asymmetric bimodal outflow condition, possibly because the left outflow was much larger than the right outflow, the left mainstream had a greater influence on the secondary mainstream on the right, and the two streams showed a small swing from left to right under the influence of each other.

**Figure 12:**
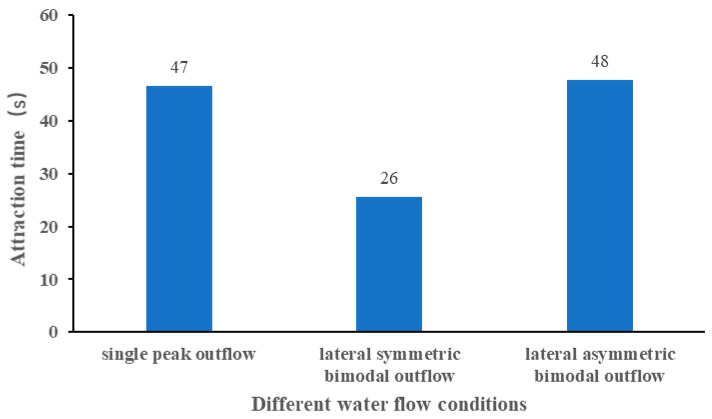
Average attraction time under different water flow conditions.

The average attraction time under different flow conditions is shown in Figure 12, and the ANOVA results are tabulated in Table 3. The shortest average attraction time was 26 s, which appeared in the lateral symmetric bimodal outflow condition, and the reason may be because the diffuse collision of the two jets weakened the flow velocity in part of the region but did not confuse the flow pattern in the region. On the other hand, it may be the main swimming mode for the middle region of the bimodal peak swimming, as the low-flow region in the middle of the bimodal peak did not hinder the swimming. The longest average attraction time was 48 s, which appeared in the lateral asymmetric bimodal outflow condition, probably because the lateral asymmetric outflow caused small oscillations of the two streams laterally, and then formed part of the unfavorable flow pattern in the swimming region.

### 3.5. Distribution at Different Time

**Figure 13:**
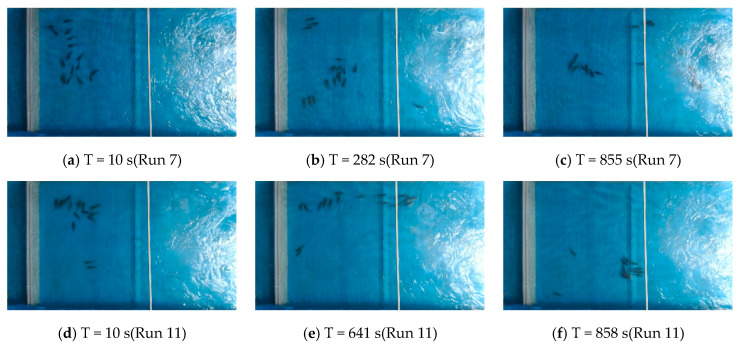

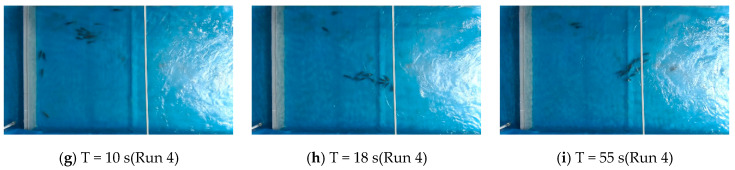
Distribution of experimental fish at different moments in some experimental groups under different water flow conditions, (**a**–**c**) run 7—lateral symmetric bimodal outflow; (**d**–**f**) run 11—lateral asymmetric bimodal outflow; and (**g**–**i**) run 4—single-peak outflow.

The distribution of some of the experimental test fish in the water tank at different moments is shown in Figure 13. Considering the short swimming time of the experimental fish in the focal observation unit (20~50 s), only a few test groups are listed here for the movement of experimental fish attracted by the jet.

The single-peak flow condition had the shortest reaction time and the best attraction effect. The experimental fish were attracted into the focal observation unit within 5 min of the start of the test for each test group; the experimental fish were attracted into the focal observation unit within 15 min of the start of the test for each of the bimodal outflow conditions. The small number of test fish that were not attracted in each test group may be due to the influence of individual factors, and these fish also did not enter the focal observation unit during the subsequent test time.

In run 7, the experimental fish were dispersed more evenly in the release unit at the beginning of the test, and when T = 855 s, there were already 15 experimental fish left in the release unit and they started to swim upstream, which successfully attracted 82% of the experimental fish to the focal observation unit. In run 11, at T = 858 s, it also successfully attracted 77% of the experimental fish to the focal observation unit. However, the fish that were attracted to the focal observation unit did not swim in large groups (more than 10 fish) as in the lateral symmetric bimodal flow condition, but in small groups of 3–5 fish, and most of them entered the focal observation unit on the right side of the low-flow attraction flow. In run 4, most of the test fish gathered in the corner in the release unit at the beginning of the trial, and 87% of the experimental fish were successfully attracted at the trial time T = 55 s. Under the single-peak outflow condition, the reaction time of the experimental fish was shorter and the attraction effect was better than that of the bimodal outflow condition.

## 4. Discussion

It was expected that by studying the rheotaxis of fish under different water flow conditions and arranging a suitable attraction flow, the entrance set of the fish passage could be improved to lure fish, thus improving the efficiency of the fish passage. Studies showed that chaotic and widely fluctuating flows discourage fish, while flows with predictability can attract fish [39]. There were significant differences in the responses of the experimental fish for the three different water flow conditions in this experiment, and Fuentes-Pérez et al. [40,41] found that hydraulic changes from power plant operation and hydrological changes in the river affect the attractiveness of the fish passage entrance to fish. Experiments by Yiqun Hou [42] also found that experimental fish had a greater tendency to select the velocity of flow for the channel than the substrate type of the channel. In addition, the number of experimental fish per group was similar to that used by researchers such as Goettel [35] and Junjun Tan [43]. There may have been a few experimental fish caught randomly for each group of experiments that participated in multiple tests, and multiple tests may have affected the experimental results. However, according to Yao Wang [38], who observed fish before and after experiments for up to one year, this effect might not be significant. Under asymmetric bimodal outflow conditions, a vortex was formed at the end of the stream of the right jet. The low-flow velocity (0.05–0.2 m/s) was still too small compared to the velocity of the fish, making the interaction time between the vortex and the fish short enough to make the fish disoriented in the vortex. Webb [44] and Cote [45] also found support for this idea. Lupandin [46] also believed that fish–vortex interactions involve time scales.

The experimental discharge was set to 80 m^3^/h to compare the behavioral responses of the experimental fish under the two bimodal outflow conditions, but the control experimental flow was 65 m^3^/h for the single-peak outflow condition. The reason was that whether comparing the induction success rate (91% > 79% > 75%), attraction success rate (82% > 39% > 34%), or fish reaction time (86 s < 391 s < 667 s), the experimental effect of single-peak outflow is better than bimodal outflow. Thus, the experimental discharge for the single-peak outflow condition was not increased to 80 m^3^/h. Enders [47] and Smith [48] suggested that fish tend to avoid flows that have wide fluctuations in flow velocity or flows that interfere with swimming trajectories on both spatial and temporal scales. This may be one explanation for the much smaller attraction success in the bimodal outflow condition than in the single-peak outflow condition. The study by Plew [49] and Chen [50] et al. provide another possible explanation that some of the test fish behaviors were influenced by school movements since carp prefer to move in groups. The reason for using *Carassius auratus* as experimental subjects in this experiment was the expectation that the fish release unit of the experimental model could be used to simulate the fish habitat, rather than simply considering the behavioral responses of individual experimental fish. Based on the qualitative test observation in the early stage, it was found that the experimental fish quickly found their preferred depth of water when entering the tank and maintained that depth to swim upstream. This is in agreement with the findings of Junjun Tan [51] and Rodriguez [52] in their experiments.

It is worth re-emphasizing that the same type of representative swimming path emerged in all three conditions, with fish swimming along the sidewalls where the flow velocity was relatively low. Some of the better swimmers swam along the mainstream, while the less competent swimmers swam along the low-flow velocity zone of the sidewalls. This is similar to the findings of Mcelroy [53]. Hang Wang [54] suggested that fish tend to swim close to the sidewalls in areas of low-flow velocity and high turbulence intensity.

Despite the time spent on experimental studies, the applicability of the obtained experimental results to practical engineering problems may still be limited to the target fish and their habitats. In addition, some sediments, rocks, and aquatic vegetation can be considered in laboratory studies to better simulate the study by arranging them according to the habitat environment of the target fish [42]. Studies on the joint mechanisms of multiple senses associated with tropism are also worth carrying out [55]. One uncertainty of the experimental study is whether the attraction flow arrangement can be extended to open water. More laboratory and field experiments are needed to validate the attraction flow arrangement with a variety of fish species of different sizes before the results obtained can be more widely applied to engineering.

To further optimize the arrangement of attraction flow, the effects of different fish attraction flow on the fish attraction effect under the single-peak outflow condition can be focused on in subsequent studies. For example, future studies can focus on the fish luring distance, sensing success rate, and attraction success rate under different discharges. Conducting research on the influence of hydrodynamic factors on fish swimming behavior and creating fish-friendly water flow conditions for fish passage projects is a key part of fish passage construction. It is important to promote the construction of fish passages, promote the development of ecohydrology, and protect the fish resources and biodiversity of rivers.

## 5. Conclusions

This study was conducted to better understand the possibility of using the rheotaxis of fish to improve fish migration efficiency near fish passage entrances or fish habitats. From the experimental results, the following main conclusions could be drawn: (1) the single-peak outflow condition not only had a higher sensing success rate for fish compared to the other two bimodal outflow conditions but also a much higher attraction success rate than the bimodal outflow conditions. (2) The fish swimming mode was not always chosen to swim against the mainstream, and the swimming mode was affected by the flow conditions and varied; under the bimodal outflow condition, the fish swimming mode was mainly the middle of the bimodal. In the single-peak outflow condition, the swimming mode was more average, and the three swimming modes could better ensure the possibility of fish swimming successfully near the entrance of the fish passage. (3) The arrangement of attraction flow was essential to improve the attraction effect in the entrance of the fish passage because the fish attracted by the flow could swim near the entrance of the fish passage in a relatively short time. The significance of this study is that it may help to improve the effect of fish attraction. It may also guide the development of biological design criteria for fish passage enhancement through hydraulic infrastructure.

## Figures and Tables

**Table 1:** Experimental runs.

Water Flow Conditions	Run No.	Number of Fish	Discharge (m^3^/h)	Water Depth (cm)	Water Outlet	
					Width Position	Vertical Position
Single-peak outflow	1	13	65	40	1/2	30 cm above the bottom
	2	12				
	3	12				
	4	15				
	5	15				
	6	13				
Lateral symmetric bimodal outflow	7	17	40 + 40	40	1/4	30 cm above the bottom
	8	16				
	9	15				
Lateral asymmetric bimodal outflow	10	17	65 + 15	40	1/4	30 cm above the bottom
	11	17				
	12	13				
	13	15				
	14	17				
	15	13				

**Table 2:** Analysis of variance (ANOVA).

	Water Flow Conditions (Mean ± Standard Deviation)			*F*	*p*
	Single Peak Outflow	Symmetrical Bimodal Outflow	Asymmetrical Bimodal Outflow		
Sensing success rate	0.90 ± 0.10	0.79 ± 0.11	0.75 ± 0.08	4.433	0.036 *
Attraction success rate	0.82 ± 0.06	0.39 ± 0.06	0.34 ± 0.13	42.557	0.000 **

* *p* < 0.05, ** *p* < 0.01.

**Table 3:** Analysis of variance (ANOVA).

	Water Flow Conditions (Mean ± Standard Deviation)			*F*	*p*
	Single Peak Outflow	Symmetrical Bimodal Outflow	Asymmetrical Bimodal Outflow		
Reaction time	86.13 ± 63.65	390.50 ± 297.94	667.32 ± 176.46	109.935	0.000 **
Attracting time	46.56 ± 31.41	25.63 ± 15.52	47.63 ± 35.68	3.817	0.025 *

* *p* < 0.05, ** *p* < 0.01.

## Data Availability

Not applicable.
